# Phylogenomic analyses reveal the diversity of laccase-coding genes in *Fonsecaea* genomes

**DOI:** 10.1371/journal.pone.0171291

**Published:** 2017-02-10

**Authors:** Leandro Ferreira Moreno, Peiying Feng, Vinicius Almir Weiss, Vania Aparecida Vicente, J. Benjamin Stielow, Sybren de Hoog

**Affiliations:** 1 CBS-KNAW Fungal Biodiversity Centre, Utrecht, Netherlands; 2 Institute of Biodiversity and Ecosystem Dynamics, University of Amsterdam, Amsterdam, Netherlands; 3 Department of Basic Pathology, Federal University of Paraná State, Curitiba, PR, Brazil; 4 Department of Dermatology, Third Affiliated Hospital, Sun Yat-sen University, Guangzhou, China; University of Minnesota, UNITED STATES

## Abstract

The genus *Fonsecaea* comprises black yeast-like fungi of clinical relevance, including etiologic agents of chromoblastomycosis and cerebral phaeohyphomycosis. Presence of melanin and assimilation of monoaromatic hydrocarbons and alkylbenzenes have been proposed as virulence factors. Multicopper oxidase (MCO) is a family of enzymes including laccases, ferroxidases and ascorbate oxidases which are able to catalyze the oxidation of various aromatic organic compounds with the reduction of molecular oxygen to water. Additionally, laccases are required for the production of fungal melanins, a cell-wall black pigment recognized as a key polymer for pathogenicity and extremotolerance in black yeast-like fungi. Although the activity of laccase enzymes has previously been reported in many wood-rotting fungi, the diversity of laccase genes in *Fonsecaea* has not yet been assessed. In this study, we identified and characterized laccase-coding genes and determined their genomic location in five clinical and environmental *Fonsecaea* species. The identification of laccases *sensu stricto* will provide insights into carbon acquisition strategies as well as melanin production in *Fonsecaea*.

## Introduction

*Fonsecaea* is a melanized fungal genus defined by sympodial conidiogenesis with conidia arranged in short chains, and in absence of budding cells. The genus affiliates to the ascomycetes order *Chaetothyriales*, comprising proven causative agents of human chromoblastomycosis and cerebral phaeohyphomycosis [1]. Among the *Fonsecaea* species, *F*. *pedrosoi* [1], *F*. *monophora* [1, 2], and *F*. *nubica* [3, 4] are the prevalent etiologic agents of chromoblastomycosis. Besides that, cerebral infection has been associated with the species *F*. *monophora* [2, 5, 6], *F*. *multimorphosa*, and *F*. *pugnacius* [7]. In addition to clinically highly significant species, *Fonsecaea* harbors a number of environmental sibling taxa [8]. *Fonsecaea erecta* and *F*. *minima* are commonly found in plant debris, while *F*. *brasiliensis* is involved in infection of cold-blooded animals [9]. The black yeast-like fungi are oligotrophic, particularly occurring in dead plants material and in low-nutrient or hydrocarbon-polluted habitats. However, the environmental niches of these fungi and the source of fungal infection of human hosts remain unclear and have not been resolved [8]. Virulence factors related to pathogenicity are similarly not fully understood, although the presence of melanin and assimilation of monoaromatic hydrocarbons and alkylbenzenes has been regarded to be significant [10]. In other pathogenic fungi, such as in *Cryptococcus neoformans* and in *Cryptococcus gattii*, melanin pigments and their synthesis from various substrates have been implicated in pathogenesis characterizing an important fungal defense against the human immune system [11, 12]. Similarly, Schnitzler *et*. *al*. (1999) showed that the fungal production of melanins and carotenoids can prevent the pathogen *Exophiala dermatitidis*, another black yeast of the order *Chaetothyriales*, from being killed in the phagolysosome by human neutrophils [13]. *Fonsecaea* species are able to produce secreted and cell-wall associated melanin which may interact with host immune cells. Melanin is believed to enhance survival in hostile environments, providing protection against UV radiation, heat, cold and against heavy metals [14].

Laccases (EC1.10.3.2) are benzenediol oxygen oxidoreductases belonging to the blue multicopper oxidase (MCO) family [15], which also includes ferroxidases, ascorbate oxidases and ceruloplasmin. Laccases catalyze the oxidation of a wide variety of phenolic compounds and aromatic amines with the concomitant reduction of molecular oxygen to water. The enzymes are widely distributed in nature, particularly in higher plants and fungi, and are involved in an extensive range of physiological functions depending on their biochemical and structural features. In fungi, laccases are responsible for pigment production, lignin degradation, sporulation, and degradation of several xenobiotics, i.e., phenols, diamines, benzenothiols and similar compounds. Fungal laccases enable the synthesis of dihydroxynaphthalene (DOPA) melanin through the oxidative polymerization of phenolic compounds leading to the production of extracellular pigments [15]. In addition, laccases have often been associated with infection of plant and animal hosts.

In general, most fungi possess multiple copies of genes encoding laccases and producing several laccase isoenzymes, which imply that these enzymes perform a variety of physiological functions. Studies on whole genome sequencing of *Exophiala dermatitidis* [16] and on transcriptomics of *F*. *monophora* [17] confirmed the abundance of laccases in black yeast-like fungi and their expression under conditions of stress. However, characterization of laccase families in these fungi requires attention in order to elucidate their exact functional relationships. The increasing availability of genomes of black yeast-like fungi has boosted research efforts in this area, and functional or comparative genomics of black-yeasts in general [18]. The present study pursues a phylogenomic analysis of five *Fonsecaea* species: *F*. *erecta*, *F*. *monophora*, *F*. *multimorphosa*, *F*. *nubica* and *F*. *pedrosoi*, in order to assess the diversity of *Fonsecaea* related laccases *sensu stricto* genes. Intraspecies variation was evaluated in two *F*. *multimorphosa* strains isolated from plant and cat respectively. Redundancy of chosen *Fonsecaea* laccases was evaluated, and their molecular characteristics, putative functions and homology models assessed. The results of this study increase our understanding of structure and diversity *Fonsecaea* laccases, and enables precise phenotype-genotype experimental approaches to better understand their physiological roles.

## Materials and methods

### Data acquisition and identification of MCOs and laccases

In total, 26 genomes and respective protein sets of black yeast-like fungi were downloaded from GenBank and used to identify laccase-coding genes (Table 1).

**Table 1:** Twenty-six black yeast-like fungi used in this study to identify laccase-coding genes.

Species	Strain number	NCBI accession number	Source
*Capronia coronata*	CBS 617.96	AMWN01000000	Decorticated wood
*Capronia epimyces*	CBS 606.96	AMGY01000000	*Pinus* wood
*Capronia semiimmersa*	CBS 273.37	JYCC01000000	Clinical chromoblastomycosis
*Cladophialophora bantiana*	CBS 173.52	JYBT01000000	Human brain abscess
*Cladophialophora carrionii*	CBS 160.54	AOFF01000000	Human chromoblastomycosis
*Cladophialophora immunda*	CBS 834.96	JYBZ01000000	Subcutaneous ulcer
*Cladophialophora psammophila*	CBS 110553	AMGX01000000	Gasolin-polluted soil
*Cladophialophora yegresii*	CBS 114405	AMGW01000000	Cactus (*Stenocereus griseus*)
*Coniosporium apollinis*	CBS 100218	AJKL01000000	Pentelic marble
*Exophiala aquamarina*	CBS 119918	AMGV01000000	*Phyllopteryx taeniolatus*
*Exophiala dermatitidis*	CBS 525.76	AFPA01000000	Human sputum
*Exophiala mesophila*	CBS 402.95	JYBW01000000	*Silicone* seal
*Exophiala oligosperma*	CBS 725.88	JYCA01000000	Human cerebral phaeohyphomycosis
*Exophiala sideris*	CBS 121828	JYBR01000000	Oak railway tie
*Exophiala spinifera*	CBS 89968	JYBY01000000	Human nasal granuloma
*Exophiala xenobiotica*	CBS 118157	JYCB01000000	Oil sludge
*Fonsecaea erecta*	CBS 125763	LVYI00000000	*Japecanga* plant
*Fonsecaea monophora*	CBS 269.37	LVKK00000000	Human chromoblastomycosis
*Fonsecaea multimorphosa A*	CBS 980.96	LVCI00000000	Cat brain abscess
*Fonsecaea multimorphosa B*	CBS 102226	JYBV01000000	Decaying palm trunk
*Fonsecaea nubica*	CBS 269.64	LVCJ00000000	Human chromoblastomycosis
*Fonsecaea pedrosoi*	CBS 271.37	JYBS01000000	Human chromoblastomycosis
*Ochroconis gallopava*	CBS 437.64	JYBX01000000	Turkey brain abscess
*Phialophora attae*	CBS 131958	LFJN01000000	Cuticle of tropical ant gynes
*Phialophora europaea*	CBS 101466	AOBU01000000	Human cutaneous infection
*Rhinocladiella mackenziei*	CBS 650.93	JYBU01000000	Cerebral phaeohyphomycosis

Phylogenetic assessment was carried out for 14 black yeast fungal strains deposited at the CBS-KNAW Fungal Biodiversity Centre (CBS), Utrecht, The Netherlands (Table 2).

**Table 2:** Isolates used in this study and their GenBank accession numbers.

Name	Strain no.	GenBank accession no.
*Cladophialophora arxii*	CBS 306.94	NR_111280
*Cladophialophora carrionii*	CBS 108.97	EU137306
*Cladophialophora devriesii*	CBS 147.84	NR_111279
*Cladophialophora emmonsii*	CBS 979.96	NR_111281
*Cladophialophora immunda*	CBS 834.96	NR_111283
*Cladophialophora minourae*	CBS 556.83	FJ225734
*Cladophialophora psammophila*	CBS 110553	NR_111183
*Cladophialophora saturnica*	CBS 118724	NR_111278
*Cladophialophora yegresii*	CBS 114405	NR_111284
*Fonsecaea brasiliensis*	CBS 119710	JN173784
*Fonsecaea erecta*	CBS 125762	KC886413
*Fonsecaea minima*	CBS 125760	KC886416
*Fonsecaea multimorphosa*	CBS 980.96	NR_111612
*Fonsecaea pedrosoi*	CBS 271.37	NR_130652

The pipeline for identification of genes belonging to the MCO family and annotation of laccases consisted of three steps: 1) Proteomes of 26 black yeast-like isolates were searched using Interproscan [19] (last accessed December, 2015) for proteins carrying the Prosite multicopper oxidase signatures PS00079 and PS00080 as well as the Cu-oxidase PFAM domains PF00394, PF07731, and PF07732; 2) Protein sequences containing at least two MCO-associated domains were aligned with plant and basidiomycete laccases and laccase, ferroxidase, and ascorbate oxidase previously identified in the fungus *Aspergillus niger* ATCC 1015 [20]; a neighbor joining phylogenetic tree was determined using Clustal Omega (http://www.ebi.ac.uk/Tools/msa/clustalo/); 3) Putative laccases were extracted from the phylogenetic tree and compared to the Laccase Engineering Database [21] (LccED, http://www.lcced.uni-stuttgart.de) using BlastP e-value cutoff 1e-10. Positive hits for putative laccases found in *Fonsecaea* species were subjected to downstream analysis.

### Annotation and manual curation of laccase coding genes

Proteins carrying the MCO signatures were rendered monophyletic with reference laccase sequences, previously identified in *A*. *niger* ATCC 1015, were searched for conserved motifs believed to be present in all laccases *sensu stricto* of ascomycetes. Initially, we assessed the composition of four specific regions composed by ungapped sequences of 8–24 amino acids, considered to be characteristic for the L1, L2, L3 and L4 domains [22], and responsible for coordinating the copper atoms in the different nuclear centers of this enzyme [23]. In addition, we investigated the presence of the conserved sequence DSG [LIV] on the C-terminus region [23, 24] and two serines and one arginine in the SDS-gate [25], a channel responsible for proton transfer.

### Structural characteristics of protein sequences

The structural characteristics of *Fonsecaea* laccases were analyzed using the programs available on the webserver Center for Biological Sequence Analysis (CBS) (http://www.cbs.dtu.dk/services/). Secreted proteins were identified using the programs SignalP Version 4.1(http://www.cbs.dtu.dk/services/SignalP/) and PrediSi (http://www.predisi.de/) for the identification of signal peptide for secretion and the putative cleavage sites. Proteins classified as intracellular were further analyzed for their subcellular location using TargetP Version 1.1(http://www.cbs.dtu.dk/services/TargetP/), iPSORT (http://ipsort.hgc.jp) and MitoProt (https://ihg.gsf.de/ihg/mitoprot.html). Isoelectric points and molecular weights were determined by ExPASy Compute pI/Mw Tool (http://web.expasy.org/compute_pi/). Putative glycosylation sites were identified using NetNGlyc 1.0 server (http://www.cbs.dtu.dk/services/NetNGlyc/).

## Results

### Identification of laccase genes

**Fig 1:**
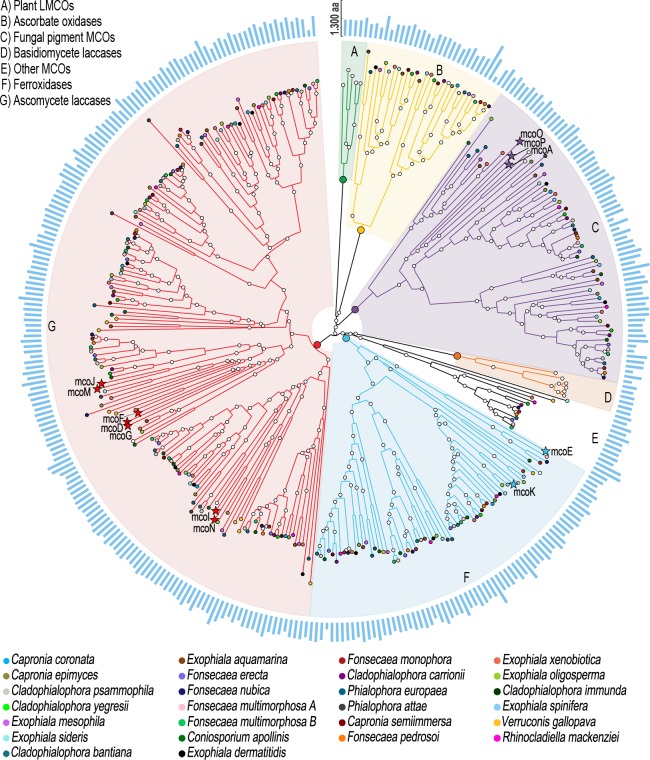
Distribution of MCOs in 26 black yeast-like fungi. Black species are indicated in different colors. The reference MCOs from *Aspergillus niger* are represented by stars and were used to identify the fungal laccase (red), ferroxidase (blue) and ascorbate oxidase (yellow) clades. The outer ring (blue) shows the variation in amino acid sequence lengths. The image was generated using the GraPhlAn package (https://huttenhower.sph.harvard.edu/graphlan).

In order to determine the number of laccase genes in the five *Fonsecaea* species, we conducted a phylogenomic study using the protein set of 26 black yeasts-like fungi (Table 1). Using Interproscan searches [19], we identified 329 proteins that possessed at least two out of five MCO associated domains: the ProSite multicopper oxidase signatures PS00079 / PS00080 and the Cu-oxidase PFAM domains PF00394, PF07731, and PF07732. *Fonsecaea* species contain different numbers of MCO genes, i.e. 14 in *F*. *monophora*, and *F*. *nubica* and 13 in the two *F*. *multimorphosa* isolates, in *F*. *pedrosoi* and in *F*. *erecta* (Fig 1 and S1 Table).

This number of MCO genes is similar with that of *A*. *niger* ATCC 1015, having 16 MCOs [20]. A Neighbor-joining phylogenetic tree was inferred using alignments of 358 MCOs protein sequences, including those 329 identified in black yeast-like fungi and 16 previously described in *A*. *niger* ATCC 1015 [20] and 2 in *Hortaea acidophila* [26], in addition to plant and basidiomycete MCOs (Table 3).

**Table 3:** Protein reference sequences of ascomycete, basidiomycete and plant MCOs.

	Organism	Protein name	NCBI accession number
**fungal MCOs**			
	*Aspergillus niger*	McoK—fungal ferroxidases	CAK97337.1
	*Aspergillus niger*	McoE—fungal ferroxidases	CAK44017.1
	*Aspergillus niger*	McoH—fungal ferroxidases	CAK37140.1
	*Aspergillus niger*	BrnA—fungal ferroxidases	CAK42098.1
	*Aspergillus niger*	McoA—fungal pigment	CAK37405.1
	*Aspergillus niger*	McoB—fungal pigment	CAK37372.1
	*Aspergillus niger*	McoC—fungal pigment	CAK47814.1
	*Aspergillus niger*	McoP—fungal pigment	CAK44915.1
	*Aspergillus niger*	McoO—fungal pigment	CAK44820.1
	*Aspergillus niger*	McoJ–laccase	CAK46289.1
	*Aspergillus niger*	McoM–laccase	CAL00426.1
	*Aspergillus niger*	McoG–laccase	CAK40046.1
	*Aspergillus niger*	McoD–laccase	CAK48347.1
	*Aspergillus niger*	McoF–laccase	CAK44895.1
	*Aspergillus niger*	McoI–laccase	CAK48652.1
	*Aspergillus niger*	McoN–laccase	CAK43464.1
	*Hortaea acidophila*	laccase I	AAY33970.1
	*Hortaea acidophila*	laccase II	AAY33971.2
**basidiomycete laccases**			
	*Pleurotus ostreatus*	Laccase	CAC69853.1
	*Pleurotus pulmonarius*	Laccase	AAX40732.1
	*Pleurotus sapidus*	Laccase	CAJ00406.1
	*Agaricus bisporus*	Laccase	ADA82243.1
	*Hypsizygus marmoreus*	Laccase	ABY78033.1
	*Lentinus sajor*	Laccase	CAD45379.1
**plant laccases**			
	*Sorghum bicolor*	Laccase	EES05524.1
	*Sorghum bicolor*	Laccase	EES05876.1
	*Sorghum bicolor*	Laccase	EES05877.1
	*Hordeum vulgare*	Laccase	BAJ98799.1
	*Zea mays*	Laccase	CAJ30499.1

Based on the phylogeny, seven monophyla were identified, with laccases arranged in a single group (Fig 1C) distinct from other MCO enzymes. The *A*. *niger* ATCC 1015 MCOs McoD, McoF, McoG, McoI, McoJ and McoM, together with the *A*. *niger* CBS 513.88 McoN (58% identical to McoI), were in the cluster that affiliates to the ascomycetes laccases. All four laccase genes previously identified in the black yeast *E*. *dermatitidis* [16] were found to cluster in the fungal laccase clade (Fig 1). In contrast, the laccase previously identified in *F*. *monophora* [17] corresponded to a ferroxidase (97% identity with Z517_00275) and thus appeared not to be a true fungal laccase. Thirty-nine protein sequences belonging to *Fonsecaea* species were extracted from the laccase cluster and compared to the LccED database, which confirmed their similarities with fungal laccases of the family B Ascomycete MCO–HFAM 4 [21]. Another cluster (Fig 1E), distinct from other MCOs, was also compared to the LccED database and revealed that the members are closely related to the Family A, Basidomycete Laccase–HFAM, which includes laccases from basidiomycetes.

To determine which of the 39 putative *Fonsecaea* laccase genes encode laccases *sensu stricto*, we accessed the presence of L1-L4 signatures, SDS-gate, C-terminus and the axial coordination, which were previously reported [23, 27] as evidence of functional laccases based on comparative analysis and crystallographic data (Table 3). The C-terminal region of the laccase AYO21_07092 in *F*. *monophora* was found missing and its corresponding genomic region was re-annotated by FGENESH 2.6 (http://www.softberry.com/) using the genomic-specific parameters available for the genus *Cladophialophora*. According to this analysis, the re-annotated protein has 591 amino, including the ProSite signature PS00080. The re-annotated AYO21_07092 is highly conserved with the laccase AYO20_08105 in *F*. *nubica* (98% BlastP identity).

We identified 26 genes possessing all characteristics of laccases including the four copper binding motifs, the L1-L4 signatures and characteristic composition of SDS-gate and the C-terminus distinctive to genes with laccase activities (Table 4 and S1 Table). These genes were classified as laccases *sensu stricto* and considered in further analyses. In both *F*. *multimorphosa* strains, a total of 5 laccases *sensu stricto* were identified and in *F*. *pedrosoi*, *F*. *monophora*, *F*. *nubica* and *F*. *erecta* 4 laccases *sensu stricto* were predicted. The number of laccase genes is known to vary among fungi in general; basidiomycetes tend to have larger numbers of laccase genes than ascomycetes. In our study, the number of laccases in *Fonsecaea* species is similar to that found in other ascomycetes. For example, *Fusarium oxysporum* contains 5 laccase-coding genes, *A*. *niger* has 6 genes[20], *H*. *acidophila* has 2 laccases genes[26]and *Trichoderma* species have 1 to 3 genes [28].

**Table 4:** Accession numbers and structural characteristics of laccases found in *Fonsecaea* spp. Putative laccase *sensu stricto* are marked with a star.

Species	ID	Length (aa)	Scaffold	# introns	GC Content %	Signal P	N-glycosylation	Asn-X-Ser/Thr	pI	MW (kDa)
***F. pedrosoi***	Z517_06970*	610	4	3	54.1	No	6-N	Yes	5.41	67.7
	Z517_06426	854	4	2	60.4	Yes	4-N	Yes	5.2	89.55
	Z517_05778*	783	4	2	54.3	Yes	3-N	Yes	5.51	82.56
	Z517_08561*	602	5	0	60.7	Yes	4-N	Yes	6.19	66.32
	Z517_07745	706	5	3	60.5	Yes	3-N	Yes	5.59	75.85
	Z517_04915*	687	3	6	54.8	No	5-N	Yes	5.44	78.58
***F. monophora***	AYO21_07092*	591	52	3	55.1	No	6-N	Yes	5.43	65.70
	AYO21_03008	855	14	2	60.5	Yes	3-N	Yes	5.16	89.62
	AYO21_07769*	786	62	2	55.1	Yes	3-N	Yes	5.51	82.77
	AYO21_05450*	602	34	0	60.8	Yes	4-N	Yes	6.19	66.35
	AYO21_06574	652	46	1	60.6	No	3-N	Yes	5.7	70.55
	AYO21_02438*	688	11	6	55.5	No	5-N	Yes	5.44	78.77
***F. nubica***	AYO20_08105*	592	61	4	54.1	No	6-N	Yes	5.17	65.61
	AYO20_02668	855	11	2	60.5	Yes	4-N	Yes	5.25	89.59
	AYO20_04230*	789	21	2	55.1	Yes	3-N	Yes	5.51	82.99
	AYO20_07966*	602	59	0	61.0	Yes	4-N	Yes	6.15	66.32
	AYO20_06102	656	37	1	61.0	No	4-N	Yes	5.69	70.75
	AYO20_00195*	689	1	6	54.6	No	5-N	Yes	5.61	78.87
	AYO20_11280	522	143	2	50.5	Y/N/T				
***F. erecta***	AYL99_03744	997	3	2	59.1	Yes	1-N	Yes	4.84	102.76
	AYL99_08651*	782	7	2	53.1	Yes	3-N	Yes	5.16	82.49
	AYL99_07449*	600	6	0	60.3	Yes	7-N	Yes	5.54	65.73
	AYL99_06775	699	5	3	60.0	Yes	4-N	Yes	5.77	75.64
	AYL99_11159*	745	13	1	56.4	Yes	5-N	Yes	5.44	79.35
	AYL99_00884*	694	1	6	54.8	No	6-N	Yes	5.7	79.42
***F. multimorphosa* A**	AYO22_09722	1279	113	2	57.6	Yes	1-N	Yes	4.36	130.05
	AYO22_04455*	799	8	2	53.9	Yes	3-N	Yes	4.95	83.95
	AYO22_03984*	603	6	0	59.7	Yes	6-N	Yes	5.33	66.05
	AYO22_06653	667	77	2	60.3	No	4-N	Yes	5.76	71.98
	AYO22_01907*	795	28	3	53.2	No	5-N	Yes	5.61	86.52
	AYO22_07432*	742	86	1	55.5	Yes	5-N	Yes	5.48	79.09
	AYO22_01881*	688	28	6	53.1	No	8-N	Yes	5.33	78.39
***F. multimorphosa* B**	Z520_10237	850	28	2	57.7	No	2-N	Yes	4.58	88.48
	Z520_04705*	799	8	2	53.9	Yes	3-N	Yes	4.95	83.95
	Z520_04209*	603	6	0	59.8	Yes	6-N	Yes	5.33	66.05
	Z520_07076	647	14	1	60.3	No	4-N	Yes	5.66	70.02
	Z520_01948*	734	3	2	53.7	Yes	4-N	Yes	5.29	79.35
	Z520_07825*	724	16	1	55.7	No	5-N	Yes	5.41	77.09
	Z520_01921*	696	3	6	53.1	No	8-N	Yes	5.33	79.21

In contrast to laccase gene clusters found in basidiomycetes [29–31], *Fonsecaea* laccase genes were randomly dispersed across the genomes (Table 4). Similar distributions have been reported in other ascomycetes, e.g. in *F*. *oxysporum* and in *Trichoderma* genomes [23, 28].

### Characteristics of *Fonsecaea* laccases *sensu stricto*

Lengths of *Fonsecaea* laccases varied between 591 to 799 amino acids, and the calculated molecular mass for the protein sequences ranges from 65.61 to 83.95 kDa with acidic isoelectric points (pI) around pH 5.2 (Table 4). These results are atypical for fungal laccases since the majority of these enzymes in fungi, with several exceptions, are 500–600 amino acid proteins ranging from 60 to 70 kDa in weight [32]. However, our results with predicted isoelectric points are in agreement with what has previously been reported for laccases of *Trichoderma* and *Fusarium* species that vary between pH 4.32–6.51 and pH 5.32–6.19 [23, 28] respectively. The G+C content of the nucleotide sequences of *Fonsecaea* laccases was considered high, ranging from 53.1% to 60.7% (Table 4), compared with an average of 53.2% of G+C content found in genes of black yeast-like fungi [18]. High G+C contents have also been reported in *Trichoderma* species [28]. A high G+C content is typical for enzymes from thermophiles and is thought to enhance stable secondary RNA structures that interfere with translation [33].

**Fig 2:**
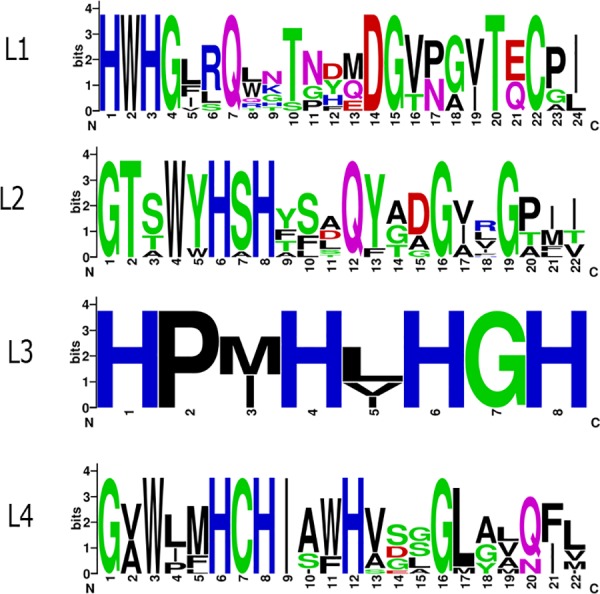
Logo sequences of L1-L4 signatures for the laccases *sensu stricto* identified in *Fonsecaea* species. Logos were generated using WebLogo (http://weblogo.berkeley.edu/).

The composition of L1-L4 motifs in *Fonsecaea* is more variable than those in other fungi. For jinstance, in L1 8/24aa (37%) are conserved, in L2 8/22 (36%), in L3 6/9 (66%), and in L4 9/22 (40%) (Fig 2).

**Fig 3:**
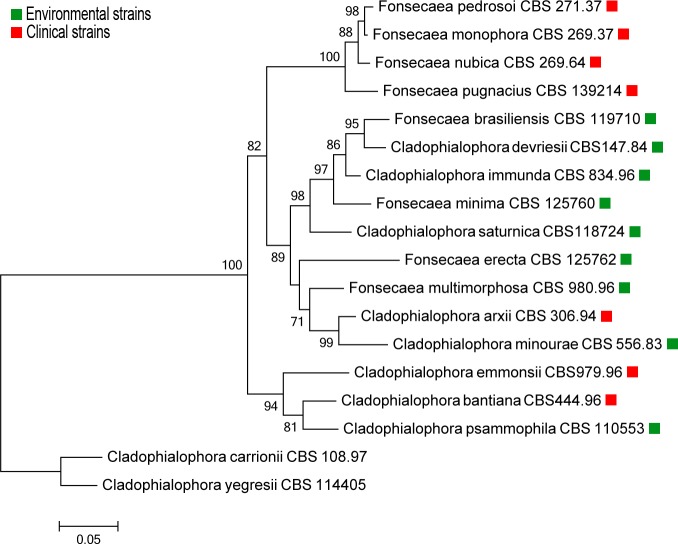
Multilocus tree of the bantiana clade based on aligned ITS and partial *BT2* sequences constructed with maximum likelihood implemented in MEGA 6.0 using the K2+G model. Bootstrap values of >70% from 1,000 resampled data sets are shown with branches. *Cladophialophora yegresii* and *C*. *carrionii* were used as outgroup. Boxes indicate environmental (green) and clinical (red) strains.

In *F*. *oxyxporum* the signatures L1-L4 are conserved for 25%, 90%, 77% and 68%, respectively [23]. These signatures are important amino acid residues of laccases due to their function in establishing the copper ion as a chelator in the center of the enzyme. The *Fonsecaea* laccase sequences contained conserved histidines as those of laccase consensus, though the conserved cysteine of the motif L2, which are regarded as classical laccases [22], were replaced by residues of threonine, glycine or alanine (Fig 2). Moreover, in the same segment, changes in the consensus QYCDGL were observed: Tyr was replaced by Phe, and Leu by Val/Ile. Similar shifts have been reported in laccases of *F*. *oxysporum* [23] and in other parts of the L2 motifs found in ascomycete fungi. For example, in the L2 of *Trichoderma* Tyr is replaced by Ala, Cys by Ser/Ala/Trp, Asp by Gly/Glu, and Leu by Val [28]. Taken together, these results suggest that the L2 region of ascomycete laccases may vary considerably, while it is suggested to be highly conserved in basidiomycetes [34]. We therefore hypothesize that *Fonsecaea* laccases have adopted various strategies to facilitate the transfer of the chelator metal ion to the trinuclear site, thus modifying its catalytic activity. In addition, other segments of the L1-L4 motifs were found altered by residues of amino acids with propensities towards similar conformations or similar hydropathic indices. For example, in segment L4, the amino acid located 10 residues downstream from the preserved Cys correspond to the axial position of copper T1. This residue is usually a Met in other MCOs; however, in the *Fonsecaea* laccases, only *F*. *pedrosoi* Z517_06970, *F*. *monophora* AYO21_07092 and *F*. *nubica* AYO20_08105 have maintained the Met residue, while in other species they are replaced by Leu (Fig 2). Note that *F*. *pedrosoi*, *F*. *monophora* and *F*. *nubica* are closely related agents of human chromoblastomycosis, while *F*. *erecta* and *F*. *multimorphosa* are mostly saprobes at some phylogenetic distance (Fig 3).

Axial coordination has an important role in redox potential (*E*_0_) of laccases [22, 35]. It has been suggested that laccase with high *E*_0_ (700–800 mV) has Leu or Phe around tenth position downstream of the conserved Cys residue in L4, whereas laccase with a Met residue has lower *E*_0_ (500 mV). Based on the axial coordination relative residue, laccases are classified in Lac1 (Met), Lac2 (Leu), and Lac3 (Phe). According to this characteristic, the putative *Fonsecaea* laccases should be classified in Lac1 and Lac2, while none of them belong to Lac3.

**Fig 4:**
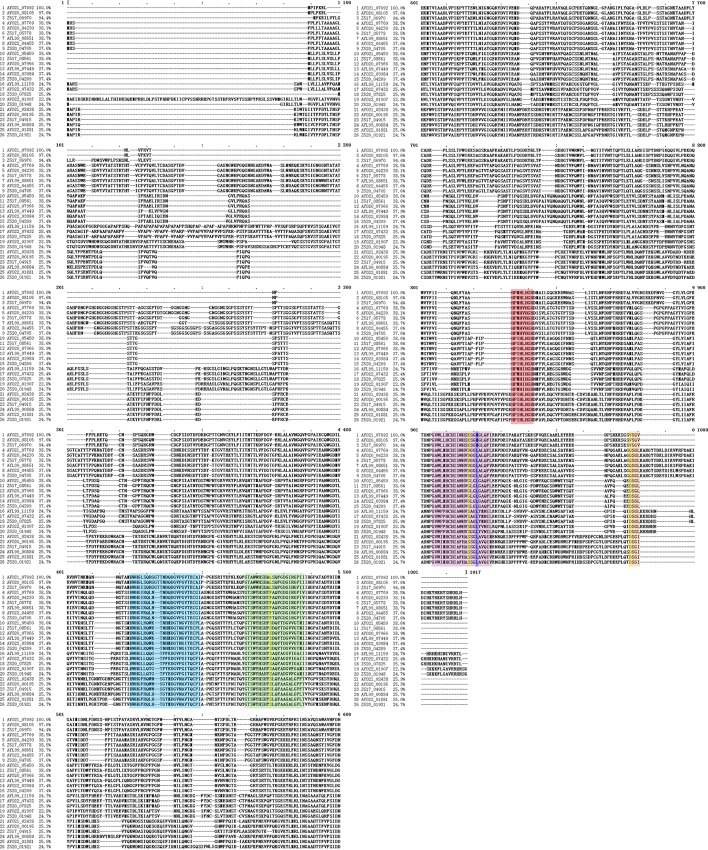
Alignment of *Fonsecae\a* laccase *sensu stricto* by MAFFT. Laccase signatures are shaded in different color: L1, blue; L2, green; L3, red; and L4, violet. The residues forming the SDS gate are shaded in yellow, and the amino acid shaded in dark blue classified the laccases as class 1 (Met) and class 2 (Leu). The conserved C-termini are in orange.

Three cupredoxin domains are present in the *Fonsecaea* laccases (S1 Table). The residues of amino acids that bind to copper to the center T1 were located in domain I, whereas the residues that coordinated coppers to the centers T2/T3 were distributed between domains I and III. The first domain from N-terminus is Cu-odidase3, then Cu-oxidase and the closest to C-terminus is Cu-oxidase2, which is a characteristic of ascomycete laccases [23]. The catalysis of laccases occurs in T1, and the electrons are transferred to the T2/T3 center, where the reduction of molecular oxygen takes place. The reduction of a dioxygen molecule to two water molecules requires four electrons and four protons. The electron transfer pathway to the trinuclear center corresponds to the preserved motif Hys-Cys-Hys located at L4, which is present in *Fonsecaea* laccases (Figs 2 and 4).

The proton transfer is assisted by the SDS-gate, which is formed by two residues of Ser and one of Asp. It is suggested that SDS-gate amino acids are conserved in ascomycete laccases, while they are absent from basidiomycete laccases. Multiple alignment with *Thielavia arenaria* laccases TaLcc1 [36] identified the SDS-gate in *Fonsecaea* laccases. With some exceptions, most of the *Fonsecaea* laccases show a conserved SDS-gate (Fig 4). The amino acid corresponding to Ser143 in TaLcc1is replaced by Leu or Phe, while the one corresponding to Ser511 is replaced by Glu, Asp or Gly and the amino acid corresponding to Asp561 is replaced by Val, Glu or Gln. Further experiments are required, to establish if these results imply that *Fonsecaea* laccases could adopt various strategies to facilitate transfer of protons to the trinuclear site, thus modifying their catalytic activity.

The C-terminus of laccase has an important role in enzyme activity. It may act as a plug that obdurate the trinuclear (T2/T3) channel, which prevents oxygen to enter the channel and water to exit. Most of the ascomycetes laccases share a conserved motif DSG[LIV] as C-terminus, which has not yet been described in basidiomycetes. It was suggested that incorrect processing of the C-terminus leads to lack of enzyme activity. The deletion of DSG[LIV] in *Melanocarpus albomyces* laccase lead to inactivation of the enzyme and lower thermostability, turnover number, and structural changes in the T2 centre [37, 38]. The conserved motif DSG[LIV] was observed in *Fonsecaea* laccases *sensu stricto*, those possessing EAG[L/I] and DD[S/A]L were regarded as false laccases (data not shown). The residue Asp in part of the *Fonsecaea* species were replaced by Val and Glu, presenting the amino acid sequences VSGV and QSGL, respectively, with or without C-termius extension, which is post-translationally removed (Fig 4). In this study, we used *A*. *niger* as model to identify the six laccases in *Fonsecaea* as *sensu stricto*. The *A*. *niger* laccases designated as Mco G, Mco J and Mco M lack a C-terminus DSGL motif. It was found that the catalytic properties of the three Mco G/J/M were hampered with limit number of oxidizing substrates compare with the others with conserved C-terminus.

Glycosylation of fungal laccases influences enzyme secretion and was suggested to plays some role in the protection of laccases from proteolytic degradation. Glycosylation is also important for catalytic center stabilization, protection against hydrolysis, copper retention, and laccase thermal stability [39]. All *Fonsecaea* laccases possess between 1 and 9 putative N-glycosylation sites, together with Asn-X-Ser/Thr sequons (Table 4).

### Phylogeny and intron position

**Fig 5:**
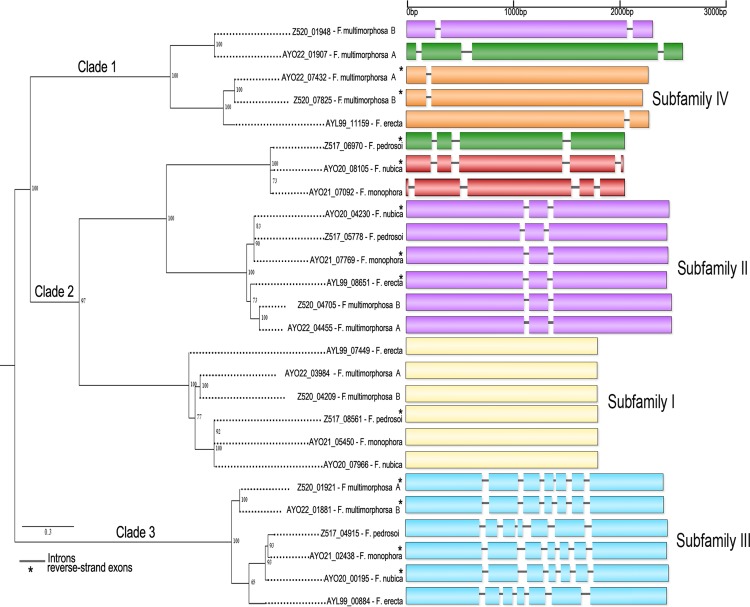
Classifications of laccases *sensu strico* based on phylogenetic analysis (left side) and abundance of introns (right side). The complete tree from 26 protein laccase sequences was built with maximum likelihood implemented in MEGA 6.0 using the K2+G model.

The number and the position of introns in laccase encoding genes in fungi have been used for classifying laccases into subfamilies, as well as for identification [29] of their distinct functions within species. For instance, seventeen laccase genes in the genome of *Coprinopsis cinerea* could be divided into two subfamilies based on intron positions [29]. In *Fonsecaea*, the density of introns in laccase *sensu stricto* genes were used to identify three distinct subfamilies present in all species analyzed (one gene per species): subfamily I with zero introns, subfamily II with 2 introns and subfamily III containing 6 introns. Two other subfamilies, which had genes with 1 or 3 introns, were restricted to *F*. *multimorphosa* and *F*. *erecta* (1 intron, subfamily IV) and *F*. *pedrosoi*, *F*. *monophora* and *F*. *multimorphosa* (3 introns, subfamily V) (Fig 5).

These introns were found distributed similarly within subfamilies. Interestingly, we observed a prevalence of gain or loss of introns in the internal region of enzymes belonging to subfamilies II and III (Fig 5). Internal regions of all laccases studied of these subfamilies contained the functional domain IPR001117 corresponding to Multicopper oxidase type 1. It has been suggested that in fungal laccases a typical substrate oxidation takes place primarily at the type 1 (T1) copper site, by abstraction of four separate electrons. Electrons are subsequently transferred to the highly conserved centers type-2 (T2) and two type-3 (T3) copper, where the reduction of molecular oxygen to water takes place [40]. Therefore we speculate that the intron–exon architecture in *Fonsecaea* laccase genes, particularly in the T1 region, could increase the diversity of this enzyme impacting its oxidative ability.

### Prediction of cellular location and possible physiological functions

The cellular location of laccases is associated with their physiological function and determines the range of substrates available for interacting with the enzyme. Most of the known fungal laccases are extracellular, although intracellular laccases have also been reported. Extracellular laccases participate in the breakdown of lignin, which is without exception true for ligninolytic peroxidases of white-rot fungi [41]. Furthermore, extracellular laccases also play an important role in the reduction of oxidative stress, recycling of organic material, and pathogenesis towards plants and animals [27, 32]. The pathogenic *Fonsecaea* species, *F*. *pedrosoi*, *F*. *monophora* and *F*. *nubica*, agents of chromoblastomycosis each have two extracellular and two intracellular laccases, while the saprobic species *F*. *erecta* has three extracellular and one intracellular laccases and both *F*. *multimorphosa* strains three extracellular and two intracellular laccases (Table 4). The putative signal peptide of the extracellular laccases corresponds to the first 17–20 residues and presents the hydrophobic region as Ala residues in position -1 and Val residues in position -3, which is relative to the cleavage site. Interestingly, all intracellular laccases contain more than three introns, while most of the extracellular laccases contain less than two introns, with the exception of Z520_07825 in *F*. *multimorphosa* CBS 102226 which lacks the signal peptide (Fig 5 and Table 4). Despite conservation of laccase *sensu stricto* motifs, similar intron positions, and phylogenetic analysis, different subcellular locations within *F*. *multimorphosa* indicates that the corresponding proteins are functionally distinct.

Unlike extracellular laccases, little is known about the activity of intracellular laccases. These isoenzymes are probably participating in the transformation of phenolic compounds in the cell, while the cell wall and spore-associated laccases are linked to the possible formation of protective cell wall compounds. Laccases associated with conidia are linked to the synthesis of pigments and other substances that protect the cell from stress factors, such as enzymatic lysis, temperature and UV light. In the human pathogenic fungus *Cryptococcus neoformans*, laccase activity is found to be involved in the membrane pigmentation and its expression constitutes a virulence factor, probably due to increase resistance to host defenses as has been proposed for other pathogenic fungi, such as *Paracoccidioides brasiliensis* [42] and *Exophiala dermatitidis* [13]. Similarly, the phytopathogenic fungus *F*. *oxysporum* possesses two intracellular laccases which may be involved in the protection of the fungus against oxidative stress and toxic compounds [43]. In these fungi, the laccases constitute part of the DOPA pathway, used to produce melanin that can confer environmental and host resistance serving as a scavenger of the free radicals produced by the oxidative system (e.g. during phagocytosis). A recent publication described the presence of three distinctive pathways in black yeasts that might allow the synthesis of melanin from various substrates [18]. The DOPA-melanin pathway, including several laccase homologs, was identified in 22 human associated-pathogens, for instance in the neurotropic species *Cladophialophora bantiana* and *Rhinocladiella mackenzie*i. The production of melanin using the DOPA pathway had previously been identified in *Fonsecaea monophora* and seems to represent an important strategy for melanogenesis in black yeast [44]. These data and other studies [45, 46] suggest that the substrates used to produce pigments in many important human pathogenic fungi could be obtained from the mammalian host, including the neurotransmitters, which partially explain the tropism for the central nervous system observed in some black yeasts, for instance in *Fonsecaea monophora* and in *Fonsecaea pugnacious*. Similar mechanism has been proposed for *Cryptococcus neoformans* [47]. It is possible that *Fonsecaea* intracellular laccases are related to any of the processes described above; however further experimental work is needed to confirm a more distinct relation between genotype and the related phenotype exhibiting metabolic features that may explain any of these functions, i.e. as described for ascomycetes yeasts in Riley et al 2016 [48].

## Conclusions

Twenty-six genes were extracted in *in silico* analysis in five clinical and environmental *Fonsecaea* species. Those genes possess features used as evidence of functional laccases, such as the multicopper oxidases functional domains, L1–L4 signatures, characteristic SDS-gate and conserved C-terminus and were assigned as laccases *sensu stricto*. A plenty of other proteins carrying the MCO domains revealed to belong to different families of copper-containing enzymes including the ascorbate oxidases and the ferroxidases. Laccases *sensu stricto* should be further investigated in order to get more insights about their possible role in fungal virulence and their biological involvement in the degradation of aromatic compounds. The identification of laccases *sensu stricto* in *Fonsecaea* species provides, to some extent, fundamental knowledge for the generation of deletion mutants for all laccase genes in studies to confirm the role of this enzyme in fungal pathogenicity, pigment production and degradation of several xenobiotics.

## Supporting information

S1 Table List of MCOs in 26 Black yeasts. (XLSX)
